# Communication between Human Dendritic Cell Subsets in Tuberculosis: Requirements for Naive CD4^+^ T Cell Stimulation

**DOI:** 10.3389/fimmu.2014.00324

**Published:** 2014-07-14

**Authors:** Laura Lozza, Maura Farinacci, Marina Bechtle, Manuela Stäber, Ulrike Zedler, Andrea Baiocchini, Franca del Nonno, Stefan H. E. Kaufmann

**Affiliations:** ^1^Department of Immunology, Max Planck Institute for Infection Biology, Berlin, Germany; ^2^Pathology Division, National Institute for Infectious Disease ‘Lazzaro Spallanzani’, Rome, Italy

**Keywords:** *Mycobacterium tuberculosis*, human, CD1c^+^ DCs, CD141^hi^ DCs, plasmacytoid DCs, CD4^+^ T cells

## Abstract

Human primary dendritic cells (DCs) are heterogeneous by phenotype, function, and tissue localization and distinct from inflammatory monocyte-derived DCs. Current information regarding the susceptibility and functional role of primary human DC subsets to *Mycobacterium tuberculosis* (Mtb) infection is limited. Here, we dissect the response of different primary DC subsets to Mtb infection. Myeloid CD11c^+^ cells and pDCs (C-type lectin 4C^+^ cells) were located in human lymph nodes (LNs) of tuberculosis (TB) patients by histochemistry. Rare CD141^hi^ DCs (C-type lectin 9A^+^ cells) were also identified. Infection with live Mtb revealed a higher responsiveness of myeloid CD1c^+^ DCs compared to CD141^hi^ DCs and pDCs. CD1c^+^ DCs produced interleukin (IL)-6, tumor necrosis factor α, and IL-1β but not IL-12p70, a cytokine important for Th1 activation and host defenses against Mtb. Yet, CD1c^+^ DCs were able to activate autologous naïve CD4^+^ T cells. By combining cell purification with fluorescence-activated cell sorting and gene expression profiling on rare cell populations, we detected in responding CD4^+^ T cells, genes related to effector-cytolytic functions and transcription factors associated with Th1, Th17, and Treg polarization, suggesting multifunctional properties in our experimental conditions. Finally, immunohistologic analyses revealed contact between CD11c^+^ cells and pDCs in LNs of TB patients and *in vitro* data suggest that cooperation between Mtb-infected CD1c^+^ DCs and pDCs favors stimulation of CD4^+^ T cells.

## Introduction

Tuberculosis (TB) is caused by the intracellular bacterial pathogen *Mycobacterium tuberculosis* (Mtb). Phagocytic cells such as macrophages engulf bacteria entering the lung and initiate a first line of defense, which controls Mtb growth and recruits pro-inflammatory cells (1). Activation of the adaptive immune responses, notably T cells, occurs only at later time points after infection and involves the migration of infected dendritic cells (DCs) to the draining lymph nodes (LNs) (2–4). Here, DCs prime naïve T cells leading to expansion and polarization of effector T cells and generation of memory T cells. Migration of DCs to LNs involves interleukin (IL)-12p40-dependent mechanisms and upregulation of CCR7 (5). Moreover, the bacterial antigens presented in the LNs need to reach a critical abundance to efficiently activate a specific CD4^+^ T cell response (4). As a corollary, inhibition of DC maturation and trafficking and, consequently, suboptimal antigen presentation, likely contribute to delayed CD4^+^ T cell responses.

Migratory and resident DCs are categorized in two main groups: myeloid (m)DCs (CD11c^+^) and plasmacytoid (p)DCs. Murine mDCs (CD11c^+^) comprise CD11b^+^ and CD11b^–^ DCs (6). After aerosol infection with Mtb, murine CD11b^+^ DCs are the major subset harboring Mtb and trafficking from the lung to the mesenteric lymph nodes (MLNs) (7). However, interferon (IFN)-γ production of CD4^+^ T cells in the MLNs seems to be mediated by non-infected CD11b^low/–^ cells rather than by CD11b^+^ DCs. Thus, so far, two unresolved questions remain to be answered: which DC subsets initiate the activation of naïve T cells in LNs and which type of T helper populations are primed in response to Mtb.

Functional specialization of DC subsets is determined by intrinsic properties such as pattern recognition receptors and external factors such as tissue localization, cytokine environment, and type of pathogen encountered. For example, in the lung, murine CD11b^+^(CD24^+^) DCs and the corresponding human homolog CD1c^+^ DCs, activate IL-17^+^ CD4^+^ T cells in response to *Aspergillus fumigatus* (8). On the contrary, human blood CD1c^+^ DCs acquire regulatory functions when stimulated with *Escherichia coli* (9). We showed that CD1c^+^ DCs produce pro-inflammatory cytokines in response to the TB vaccine Bacille Calmette–Guérin (BCG) and low levels of IL-10 (10).

Murine splenic CD11b^–^ CD8α^+^ DCs and non-lymphoid tissue CD11b^–^ CD103^+^ DCs are highly related to human CD141^hi^ DCs (11). CD11b^–^ CD8α^+^ DCs are susceptible to *Listeria monocytogenes* infection and their depletion enhances host defense (12, 13). CD141^hi^ DCs are well characterized for cross-presentation and for their ability to present necrotic antigens by mean of C-type lectin 9A (CLEC9A) (14–16). This complex network of DC subsets emphasizes differential susceptibility of distinct DC populations to pathogens and pathogen-associated molecular patterns.

In addition, distinct DC subsets may communicate during infection to promote or inhibit T cell responses (17). Cross-talk of mDCs and pDCs promotes cytotoxic T cell activation and IL-12 production in response to herpes simplex virus or TLR9 ligand (18–20). Besides the crucial role of pDCs in viral infection (21), we have shown that pDCs are activated by BCG-infected CD1c^+^ DCs and enhance BCG-specific CD8^+^ T cell responses independently of TLR9 and type I IFN. Thus, cooperation of mDCs and pDCs occurs during bacterial infection.

We embarked on the characterization of human DC responses to Mtb infection by visualizing DC subsets in LNs of TB patients. We determined the responsiveness of CD1c^+^ DCs, CD141^hi^ DCs, and pDCs to live Mtb infection and their ability to stimulate autologous naive CD4^+^ T cells.

## Materials and Methods

### Media and reagents

For FACS sorting (FACS Aria II, BD Biosciences) and acquisition (FACSCanto II equipped with FACSDIVA Software, BD Biosciences) the following anti-human antibodies were used: BDCA-1-FITC, BDCA-4-PE, and BDCA-3-APC from Miltenyi Biotec; CD3 Alexa Fluor 700 (UCHT1), CD4 Pacific Blue (RPA-T4), CD11c Alexa Fluor 700 (B-ly6), CD14 Pacific Blue (M5E2), CD56 Pe-Cy7 (B159), and Annexin V APC from BD Biosciences; CD25 Alexa Fluor 488 (VT-072), CD19 Pe-Cy5 (HIB19), CD20 PerCP (2H7), CD45RA Alexa Fluor 700 (HI100), and HLA-DR BV 570 (L243), from Biolegend; CD123 eFluor450 (6H6) and Propidium Iodide (PI) from eBioscience; and CD40 PE (82111) and CD127 PE (40131) from R&D Systems. Cultures were performed using complete RPMI media 1640 (Life Technologies) in the presence of 5% human serum (Lonza) without antibiotics.

### Cell isolations

Experiments with donor material were approved by the Ethics Committee of the Charité University Hospital (Charité Universitätsmedizin) in Berlin, Germany [EA2/064/14]. Donations received from blood bank donors were anonymized. DCs were isolated from buffy coats obtained from the German Red Cross blood bank (DRK-Blutspendedienst Ost) by Ficoll–Hypaque gradient (Biochrom), as described previously (10). Briefly, DCs were enriched from PBMCs by MACS separation using BDCA-1-FITC, BDCA-4-PE, and BDCA-3-APC followed by incubation with FITC-, PE-, and APC-beads. The positive fraction was stained with lineage markers (lin) (α-CD3, α-CD19, α-CD20, α-CD56, α-CD14) and α-HLA-DR and DCs were further purified by cell sorting according to the following staining: pDCs (lin^−^HLA-DR^+^BDCA-4^+^BDCA-1^−^BDCA-3^−^), CD1c^+^ (=BDCA-1^+^) mDCs (lin^−^HLA-DR^+^BDCA-4^−^BDCA-1^+^BDCA-3^–^), and CD141^hi^ (=BDCA-3^+^) mDCs (lin^−^HLA-DR^+^BDCA-4^−^BDCA-1^−^BDCA-3^+^). Naïve CD4^+^ T (CD3^+^) cells were enriched using MACS beads (Miltenyi Biotec) followed by sorting according to naïve (CD45RA^+^CD127^high^CD25^–^) and T cell markers. Sorted cells with purity higher than 98% were used for experiments.

### Cell culture conditions

A total of 25,000 CD1c^+^ DCs, CD141^hi^ DCs, or pDCs were infected using virulent Mtb strain (H37Rv) expressing GFP at an MOI of five. After 2 h, extracellular bacteria were removed by extensive washing and cells were cultured for another 16 h unless otherwise indicated. In co-culture conditions, unstimulated pDCs were added to Mtb-infected mDC cultures, 2 h post-infection, at a 1:1 ratio and supernatants were harvested after 14 h. In some conditions, CD1c^+^ DCs were stimulated with 100 ng/mL Lipopolysaccharide (LPS; Sigma-Aldrich) and 2.5 μg/mL Resiquimod (R848; Invitrogen) for 16 h.

To study naïve CD4^+^ T cell proliferation, 250,000 naïve CD4 T cells were stimulated at a 1:10 ratio (mDC subset:T cell) with autologous DCs previously infected for 16 h with Mtb. Proliferation was visualized after 7 days by carboxyfluorescein succinimidyl ester (CFSE) dilution.

### Flow cytometric analysis

After Mtb infection or TLR stimulation, DCs were harvested and labeled with α-hCD123 and α-hCD11c antibodies that allow distinction between pDCs (CD123^high/low^CD11c^–^) and CD1c^+^ DCs (CD123^low/–^CD11c^+^) in co-culture conditions. α-hCD141 was used to label CD141^hi^ DCs. Apoptotic cells were detected as Annexin V^+^ PI^–^ (early apoptotic cells) or Annexin V^+^ PI^+^ (late apoptotic cells). Necrotic cells were detected as PI^+^ cells. Mean fluorescence intensity of HLA-DR, CD40, and CD83 was showed after subtraction from baseline values of unstimulated conditions. Naïve CD4^+^ T cells were labeled with CFSE according to manufacturer’s instructions (Molecular Probes) and proliferation analyzed after 7 days of culture with autologous DCs. Proliferating cells were gated as CD4^+^CD3^+^CFSE^low^ cells. Analysis was performed using FlowJo (TreeStar).

### ELISA

Unless otherwise indicated, ELISA was performed at 16 h post-stimulation (2 h infection and additional 14 h incubation). IFN-α was measured by VeriKine Human Interferon Alpha ELISA Kit (PBL Interferon Source), granzyme B (GrB) by PeliKine Compact Human Granzyme B Elisa Kit (Sanquin), IL-1β and IL-12p70 by R&D Systems, tumor necrosis factor (TNF)-α, and IL-6 by Ready-SET-Go kit (eBioscience).

### RT-PCR

Gene expression levels were analyzed simultaneously using the 96.96 Dynamic Array Integrated Fluidic Circuits (IFCs) from Fluidigm. After 7 days of proliferation, CD4^+^CD3^+^CFSE^low^ cells were sorted in triplicates of 100 cells and collected in a 96-well PCR plate (Eppendorf). The genes of interest were pre-amplified using a mix of TaqMan Gene expression Assays (Applied Biosystems). The cDNA and the single TaqMan assays were then loaded in a microfluidic chip using 96.96 IFC Controller HX according to manufacturer’s protocol. Quantitative PCR was performed with the BioMark™ HD System (Fluidigm). Data were exported with the Real-time PCR Analysis Software (Fluidigm) and analyzed with Microsoft Office Excel. ΔCt was referred to *GAPDH* (NM_001256799.1) transcript. The threshold for ΔCt calculation was set at Ct < 30 and Ct values >30 were excluded. To compare data from different donors and chips, fold change in transcripts (2^–[(ΔCt)reference−ΔCt(value)]^) was calculated relative to ΔCt of CD4^+^CD3^+^CFSE^low^ cells stimulated with Mtb-infected CD1c^+^ DCs (ΔCt_reference_). The following transcripts were analyzed: *GZMB* (NM_004131.4), *PRF1* (NM_000594.3), *IRF4* (NM_001195286), *CXCR3* (NM_001504), *CCR7* (NM_001838), *TBX21* (NM_013351), *RORC* (NM_001001523), *GATA3* (NM_001 002295), and *FOXP3* (NM_001114377).

### Immunofluorescence

Formalin-fixed paraffin-embedded tissue blocks of LN specimens from TB^+^ HIV^–^ patients with pulmonary TB were obtained from the Lazzaro Spallanzani National Institute for Infectious Diseases (INMI), Translational Research Unit, Department of Epidemiology and Preclinical Research, Rome, Italy. Specimens belong to archived autopsies of patients with pulmonary TB and were *Mtb* culture-positive or had positive stains for acid-fast bacilli. In addition, LN tissue slides from patients with pulmonary TB were obtained from Bio-Cat GmbH, Heidelberg, Germany.

Lymph node tissues from subjects without TB but undergoing cancer screening (Bio-Cat GmbH, Heidelberg, Germany) were used as comparison group. Paraffin-embedded sections of 5-μm thickness were deparaffinized and rehydrated. Epitopes were heat-retrieved in a pressure cooker with Target Retrieval Solution, High pH (Dako), and tissue sections were blocked with 1% horse serum (PAA technologies), 5% donkey serum (PAA technologies), 5% sheep serum, and 1% bovine serum albumin (Sigma–Aldrich) in PBS with 0.05% Tween-20 (PBS-T) at room temperature for 45 min. Goat polyclonal antibody (Ab) against CLEC4C, sheep polyclonal Ab against CLEC9A (R&D Systems), rabbit polyclonal Ab against CD3 (Dako), CD20 and CD11c (Abcam), mouse monoclonal Ab against GrB (Monosan), were applied to tissue sections at 4°C, overnight. After three washes with PBS-T, sections were incubated with NL™ 557 donkey anti-goat or anti-sheep Ab (R&D Systems) at room temperature for 45 min, washed and then incubated with Alexa Fluor 647 goat anti-mouse and Alexa Fluor 488 goat anti-rabbit (Invitrogen) at room temperature for 45 min. After PBS-T wash, nuclei were stained with DAPI (Sigma) and tissue sections were mounted in Confocal Matrix (Imm Tech). Images were captured by a Leica DMR epifluorescence microscope equipped with Nikon Digital DX M1200F.

### Statistical analysis

We performed statistical analysis using Graph Pad Prism 5 Software. Group data were tested for normal distribution (Shapiro–Wilk normality test). Wilcoxon signed-rank or Mann–Whitney tests were used for paired or unpaired observations, respectively. Analysis of Variance (ANOVA) was used to compare more than two sets of data. Lines and error bars represent mean ± SD. For the increase of GrB and IFN-α concentrations, significance of the interaction between treatment groups and time points was tested using permutation test from the R package lmPerm version 1.1.

## Results

### Distribution of pDCs and mDCs in LNs of TB patients

**Figure 1:**
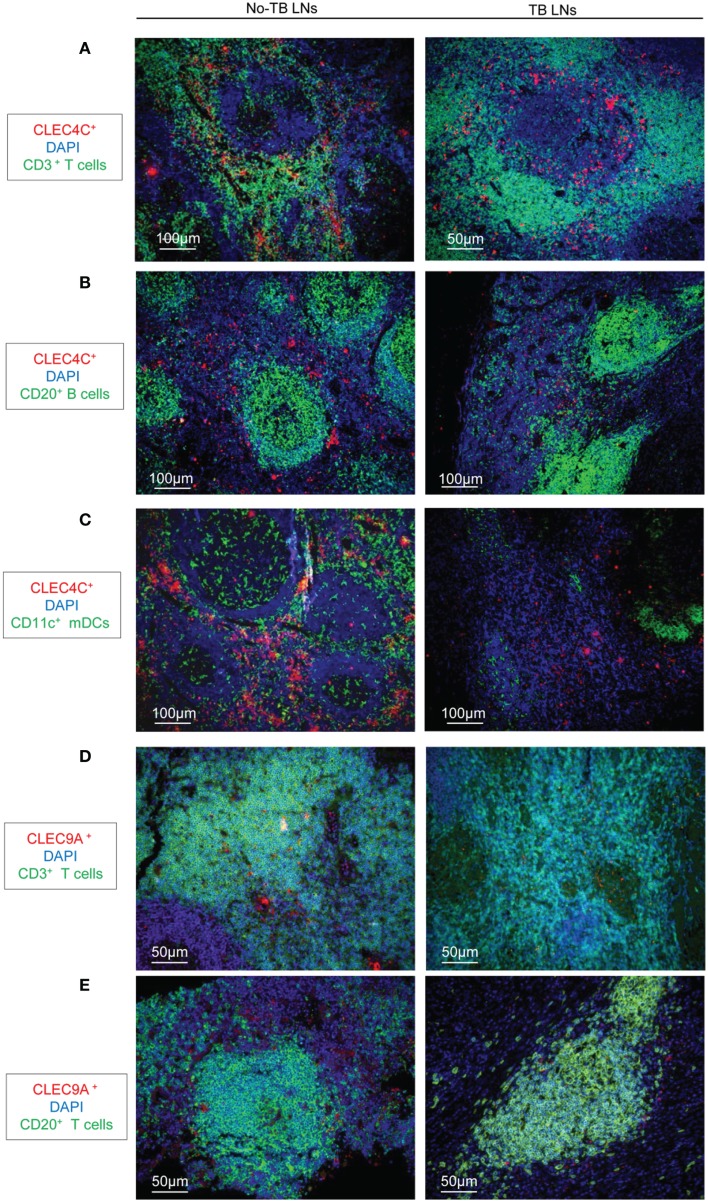
**Immunostaining of DCs in LNs from TB patients and individuals without TB**. **(A–C)** Immunostaining of pDCs (CLEC4C, red) and **(A)** T cells (CD3^+^, green); **(B)** B cells (CD20^+^, green); **(C)** CD11c^+^ cells (green). DAPI (blue) indicates cell nuclei. **(D,E)** Immunostaining of CD141^hi^ DCs (CLEC9A, red); and **(D)** T cells (CD3^+^, green); or **(E)** B cells (CD20^+^, green). DAPI (blue) indicates cell nuclei. Magnification 10× or 20×. Left: reactive LNs from individuals without TB (no-TB LNs); right: LNs from TB patients (TB LNs). One representative experiment out of three controls (no-TB) and four TB cases shown.

**Figure 2:**
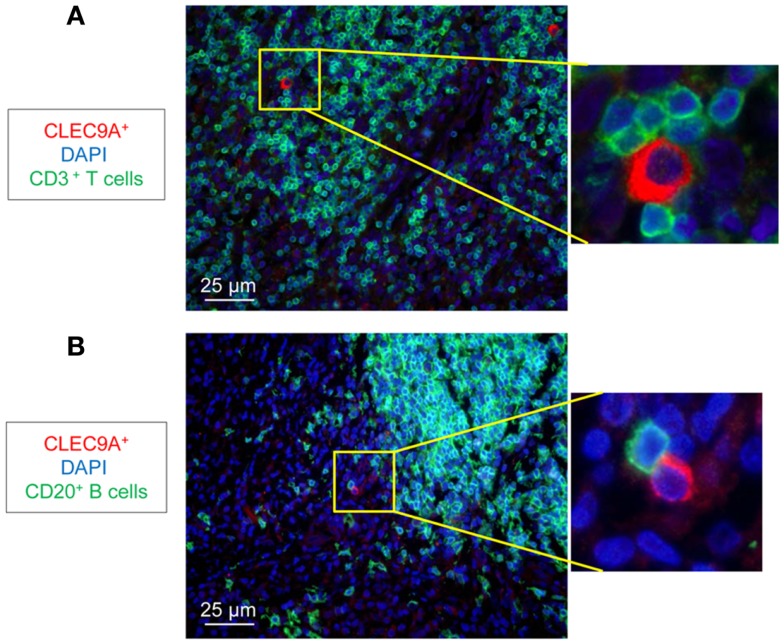
**Identification of CD141^hi^ DCs in LNs of TB patients**. Immunostaining of CD141^hi^ DCs (CLEC9A) with **(A)** T cells (CD3^+^, green) and **(B)** B cells (CD20^+^, green). DAPI (blue) indicates cell nuclei. Magnification 40×. One representative experiment out of three TB cases shown.

We analyzed the distribution of DC populations in reactive LNs of individuals without TB (no-TB) and TB patients. In both groups, abundant pDCs (identified by CLEC4C staining) were detected in close vicinity to CD3^+^ T cells (Figure 1A) but were rare in B cell areas (Figure 1B). CD11c^+^ cells, including mDCs, were regularly distributed in LNs of individuals without TB while some clusters of cells were detected in TB patients (Figure 1C). Among mDCs, CD141^hi^ DCs (identified by CLEC9A staining) were found in LNs of no-TB individuals within CD3^+^ and CD20^+^ cell areas (Figures 1D,E, left) but were rare in LNs of TB patients (Figures 1D,E, right). At higher magnitude single CLEC9A cells were clearly distinguishable and located in contact with CD3^+^ or CD20^+^ cells in TB patients (Figures 2A,B).

### Contact between mDCs and pDCs and release of GrB by pDCs in LNs of TB patients

**Figure 3:**
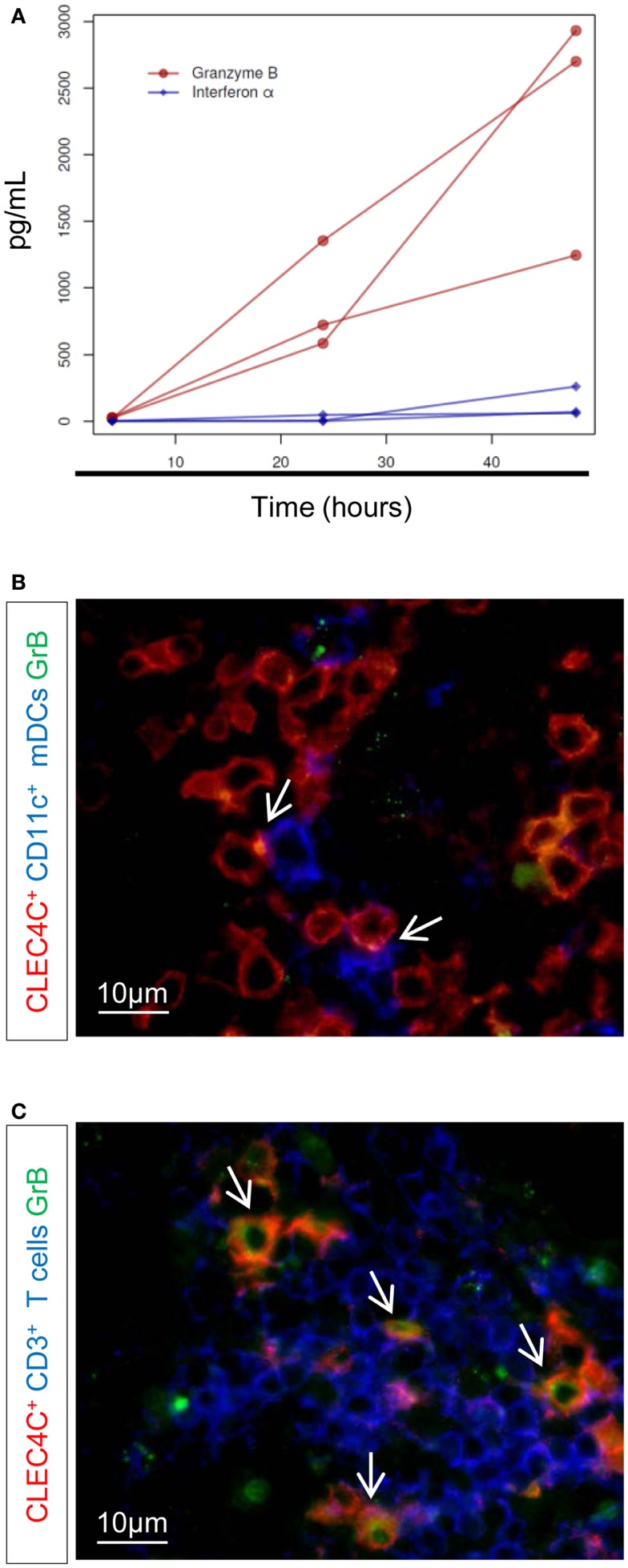
**Production of IFN-α and GrB after Mtb infection**. **(A)** ELISA of IFN-α (blue diamonds) and granzyme B (GrB) (red circles), at 4, 24, and 48 h after infection with Mtb. Only results from Mtb-infected CD1^+^–pDC cultures are shown (no detectable concentration of either GrB or IFN-α was found in Mtb-infected CD1c^+^ DC monocultures). Three donors in one experiment are shown (permutation test). Immunostaining of **(B)** contact area between CD11c^+^ cells (blue) and pDCs (CLEC4C, red) and **(C)** GrB^+^-producing pDCs (in red) or GrB^+^-producing CD3^+^ (in blue). GrB is stained in green. Arrows indicate contact area **(B)** or CLEC4C–GrB co-staining **(C)**. Magnification 100×. One representative experiment out of three TB cases shown.

We previously described that pDCs help CD1c^+^ DCs in the control of BCG infection and induction of mycobacteria-specific CD8 T cell response(s). In this context, pDCs produced GrB in high abundance but not type I IFN (10). We determined whether Mtb induces a similar cross-talk between pDCs and CD1c^+^ DCs. In contrast to BCG stimulation (10), low concentrations of IFN-α were detected in culture of pDCs with Mtb-infected CD1c^+^ DCs (MOI 5, 48 h post-infection) (Figure 3A, blue diamonds, *p* < 0.05 compared to Mtb-infected CD1c^+^ DCs monocultures). However, GrB production remained predominant (Figure 3A, red circles, *p* < 0.001 compare to Mtb-infected CD1c^+^ DCs monocultures) indicating that pDC response to mycobacterial infection is skewed toward GrB production and regulated by the state of activation of infected mDCs rather than by the type of microorganism (pathogenic Mtb vs. attenuated BCG) (10). Similarly, *ex vivo* co-staining of LNs of TB patients revealed discrete contact areas between CD11c^+^ DCs and pDCs (Figure 3B), as well as between CD3^+^ cells and pDCs (Figure 3C) and the presence of GrB^+^ pDCs (Figure 3C). This spatial distribution and functional capacity of pDCs in LNs points to a cross-talk between pDCs and mDCs during active TB.

### Mtb-infected CD1c^+^ DCs induce naïve CD4^+^ T cell proliferation, which is enhanced by pDCs

**Figure 4:**
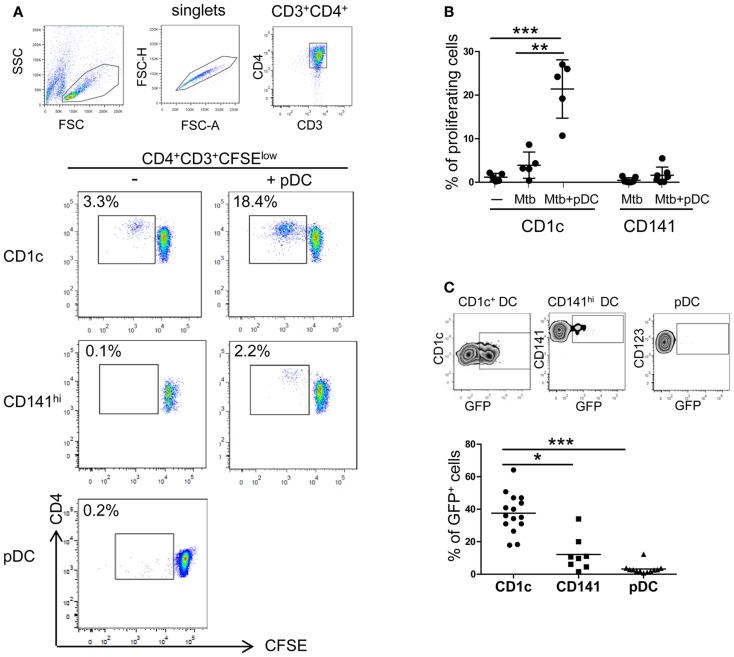
**CD4^+^ T cell activation by CD1c^+^ DCs**. **(A)** and **(B)** Naïve CD4 T cells were labeled with CFSE and proliferation was measured after 7 days of culture in the presence of autologous CD1c^+^ DCs ± pDCs (CD1c), CD141^hi^ DCs ± pDCs (CD141^hi^), or pDCs alone (pDC) previously stimulated with live Mtb for 16 h. DC:T cell ratio was 1:10. **(A)** One representative flow cytometric CD4 T cell staining shown; lymphocytes were gated according to morphological parameters and doublets excluded. Proliferating CD4^+^ T cells were gated as CD4^+^CD3^+^CFSE^low^ cells. **(B)** Mean ± SD of proliferating CD4^+^ T cells in response to CD1c^+^ DCs ± pDCs or CD141^hi^ DCs ± pDCs of at least five donors in two independent experiments. **(C)** One representative gating strategy of Mtb-infected CD1c^+^ DCs (top left), CD141^hi^ DCs (top middle), and pDCs (top right) and mean ± SD of Mtb-infected cells of at least eight donors in four independent experiments (bottom). Cells were infected at MOI 5 with Mtb-expressing GFP and stained for specific DC marker after 16 h. Infected cells were measured as percentage of GFP^+^ cells. Kruskal–Wallis ANOVA; *p < 0.05, **p < 0.01, ***p < 0.001.

To gain deeper insights into DC subset interplay during Mtb infection, CD1c^+^ DCs, CD141^hi^ DCs, and pDCs were isolated from peripheral blood of healthy donors. The subsets were cultured with virulent Mtb-expressing GFP and analyzed for their ability to prime naïve CD4^+^ T cells. Autologous naïve CD4^+^ T cells proliferated in response to infected CD1c^+^ DCs but not to CD141^hi^ DCs and pDCs (Figures 4A,B). These differences could be due to lower ability of CD141^hi^ DCs and pDCs to phagocytose whole bacilli (Figure 4C) and consequently, to present antigens to T cells.

Proliferation of autologous naïve CD4^+^ T cells in response to Mtb-infected CD1c^+^ DCs was enhanced by the presence of pDCs (Figures 4A,B). A similar trend was observed when pDCs were cultured with Mtb-infected CD141^hi^ DCs although naive CD4^+^ T cells were more responsive to infected CD1c^+^ DCs (Figures 4A,B).

**Figure 5:**
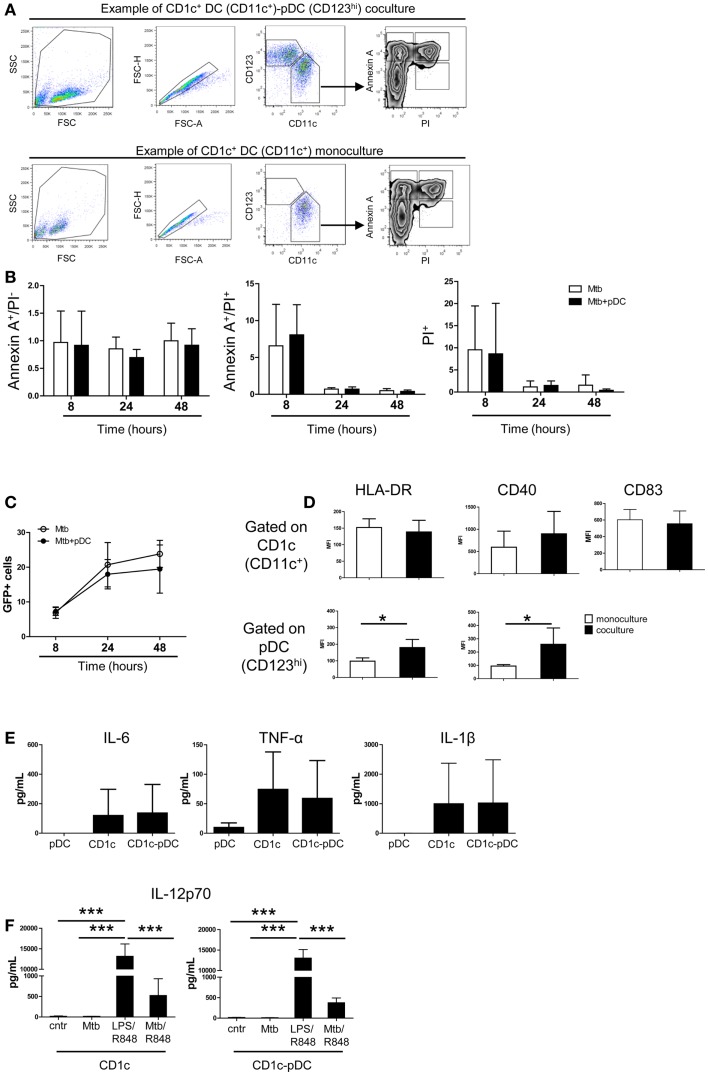
**Survival, infection and cytokine production by CD1c^+^ DCs is not affected by pDCs**. **(A)** One representative gating strategy used to visualize DC subsets and apoptotic–necrotic CD1c^+^ DCs. After exclusion of doublets, pDCs were gated as CD123^high^ CD11c^–^ cells and CD1c^+^ DCs as CD123^low/–^CD11c^+^ cells. CD1c^+^ DCs were then gated as early apoptotic cells (Annexin A^+^ PI^–^), late apoptotic cells (Annexin A^+^ PI^+^), and necrotic cells (PI^+^). **(B)** Relative number of apoptotic and necrotic CD1c^+^ DCs at different time points after Mtb infection in the presence (Mtb + pDCs) or absence of pDCs (Mtb). The number was obtained by normalizing the percentages of apoptotic–necrotic CD1c^+^ DCs after infection to the percentages of the respective controls (CD1c^+^ DCs ± pDCs in the absence of Mtb). **(C)** Percentage of Mtb-infected (GFP^+^)-CD1c^+^ DCs in the presence or absence of pDCs. **(D)** Mean fluorescence intensity of HLA-DR, CD40, and CD83 in Mtb-infected mono- (white bars) or co-cultures (black bars) gated on CD1c^+^ DCs (top) or pDCs (bottom panel) (Mann–Whitney test). **(E)** Cytokine production by pDCs, CD1c^+^ DCs, or CD1c-pDC cultures 16 h post-infection. **(F)** Production of IL-12p70 by CD1c^+^ DC or CD1c^+^ DC-pDC cultures 16 h post-infection in the absence (Mtb) or in the presence (Mtb/R848) of TLR7/8 ligands or after stimulation with TLR4 and TLR7/8 ligands (LPS/R848). Control (Cntr) indicated unstimulated cells. Data are obtained from six donors in two independent experiments. One-way ANOVA; *p < 0.05, ***p < 0.001.

We then focused on the CD1c^+^ DC and pDC interaction. Apoptosis of CD1c^+^ DCs was not affected by pDCs excluding that pDCs killed CD1c^+^ DCs through type I IFN or GrB-mediated mechanisms (22, 23) (Figures 5A,B). Similarly, pDCs did not affect the number of Mtb-infected CD1c^+^ DCs (Figure 5C). Contact with Mtb-infected CD1c^+^ DCs elicited higher expression of HLA-DR and CD40 but not of CD83 on the surface of pDCs. (Figure 5D, bottom and data not shown). After infection, CD1c^+^ DCs expressed HLA-DR, CD40, and CD83 (Figure 5D, top) and produced IL-6, TNF-α, and IL-1β but not IL-23, TGF-β, or IL-12p70 (Figures 5E,F and data not shown). We excluded a functional defect in CD1c^+^ DCs since IL-12p70 was produced in the presence of LPS (TLR4 ligand) and R848 (TLR7/8 ligands) (24) and partially induced in infected cells triggered with R848 (Figure 5F). These data identify CD1c^+^ DCs as the most responsive DC subset to Mtb infection but also highlight their dependency on additional stimuli for optimal IL-12 production. Moreover the data reveal that the presence of pDCs favors CD4^+^ T cell expansion.

### CD4^+^ T cells activated by Mtb-infected DCs upregulate cytolytic functions and display diverse polarization

**Figure 6:**
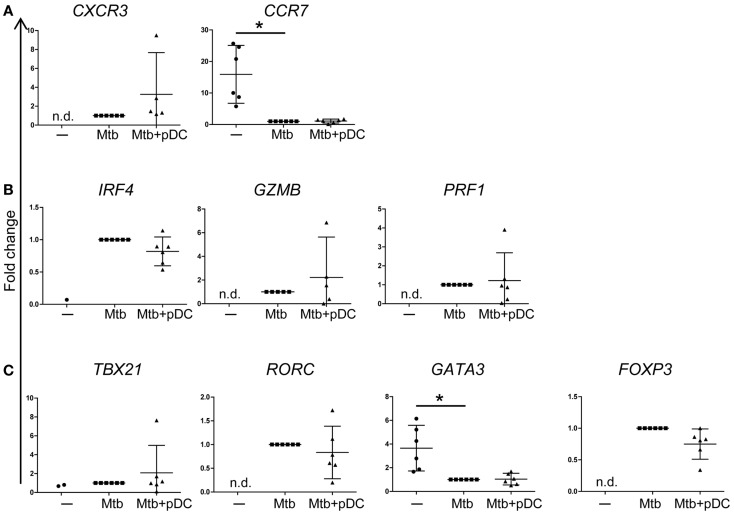
**Gene expression profile of CD4^+^ T cells**. Fold changes of **(A)** *CXCR3*, *CCR7*, **(B)** *IRF4*, GrB (*GZMB*), perforin (*PRF1*), **(C)** Tbet (*TBX21*), RORγT (*RORC*), *GATA3*, and *FOXP3* transcripts, measured in CD4^+^CD3^+^ CFSE^low^ cells, in the presence (Mtb + pDC) or absence of pDCs (Mtb) and unstimulated CD4^+^CD3^+^ cells (−). The threshold for ΔCt calculation was set at Ct < 30 and Ct values >30 were excluded. ΔCt was calculated to GAPDH. To compare data from different donors and chips, fold change in transcripts (2^−[(ΔCt)reference−ΔCt(value)]^) was calculated relative to ΔCt of CD4^+^CD3^+^CFSE^low^ cells stimulated with Mtb-infected CD1c^+^ DCs (ΔCt_reference_) since most of the transcripts were undetectable in unstimulated conditions (−). In unstimulated conditions (−), IRF4 and Tbet transcripts were detectable only in one and two samples out of six, respectively. Mean ±95% confidential interval of at least five donors in two independent experiments shown. Wilcoxon signed-rank test; *p < 0.05; n.d., non-detectable.

To characterize the phenotype of responding CD4^+^ T cells, we performed gene expression profiling of sorted CD3^+^CD4^+^ CFSE^low^ T cells by using a protocol that allows gene expression analysis of rare populations (100 cells). CD4^+^ T cell expansion was associated with upregulation of CXCR3 and downregulation of CCR7 gene expression (Figure 6A) consistent with a phenotype of activated T cells. In addition, proliferating CD4^+^ T cells upregulated the expression of IRF4, GrB (*GZMB*), and perforin (*PRF1*) suggesting that they express cytolytic functions (Figure 6B). Interestingly, gene expression profiling of CFSE^low^ CD4^+^ T cells revealed upregulation of Tbet (*TBX21*), RORγt (*RORC*), and FOXP3 but not GATA3 transcripts (Figure 6C). The presence of pDCs did not modulate the gene expression profile of these transcription factors (Figures 6A–C). We conclude that effector CD4^+^ T cells activated by Mtb-infected CD1c^+^ DCs are heterogeneous and potentially polarized toward Th1, Th17, Treg cells but not Th2 cells.

## Discussion

Dendritic cells are important players in the early phase of Mtb infection mostly by modulating the activation of T lymphocytes (25). The heterogeneity of DC subsets indicates a division of labor during infection that could impact on the quality of the T cell response. The role of primary DC subsets in Mtb infection, particularly in human TB, is incompletely understood. Here, we identify both mDCs and pDCs in LNs of TB patients. Few CLEC9A^+^ cells, the marker used to identify CD141^hi^ DCs (15, 26, 27), are also found in T and B cell areas.

We demonstrate that CD1c^+^ DCs engulf and respond to live Mtb more efficiently than CD141^hi^ DCs and pDCs. After infection, they upregulated HLA-DR and CD40, which are required for CD4^+^ T cell priming (28). CD1c^+^ DCs produced pro-inflammatory cytokines, but not IL-12p70. Optimal production of IL-12 by CD1c^+^ DCs has been found to depend on TLR 4/7/8 triggering and to be promoted by IFN-γ or CD40L (24, 29, 30). Moreover, macrophages were found to require a prime signal by IFN-γ to produce IL-12 in response of Mtb (31). Thus, it is likely that the lack of IL-12 is due to absence of adequate stimuli. It is known that Mtb interferes with macrophage and DC activation by modulating cytokine production and MHC class II expression (32–34). Recognition of mannosylated lipoarabinomannan (ManLam) by DC-SIGN also inhibits monocyte-derived (mo)DC functions and induces IL-10 (35, 36). Primary human DCs do not express DC-SIGN or mannose receptor (37, 38) and whether Mtb actively inhibits IL-12 signaling in primary CD1c^+^ DCs needs further investigation.

The inhibition of moDC functions is not absolute since moDCs still produce cytokines and induce Th1 cells (39, 40). Similarly, we found that CD1c^+^ DCs are still able to stimulate naïve CD4^+^ T cells to become effector T cells. Furthermore, bacterial numbers could affect functions of DCs, and consequently T cell activation: low numbers may delay T cell responses (4, 41), whereas high numbers of bacilli could inhibit DC function or induce T cell exhaustion (42). To understand the physiological state of activation of CD1c^+^ DCs and their antigen presentation capacity, a closer look at these cells in LNs or lungs of Mtb-infected individuals is essential albeit limited by scarce availability of human tissues.

We found that CD141^hi^ DCs fail to directly activate naïve CD4^+^ T cells. However, mice lacking essential transcription factors for CD8α^+^ DC and tissue CD11b^−^CD103^+^ DC development are susceptible to Mtb infection (43, 44). Although these transcription factors influence the functions of other cell types, these studies suggest that CD8α^+^ and CD11b^−^CD103^+^ DCs are indeed involved in protection against TB. Since localization and cytokine environment affect DC function, it is likely that immature blood CD141^hi^ DCs respond less efficiently than LN-resident CD141^hi^ DCs to Mtb infection in the absence of additional stimuli. In fact, when properly stimulated with TLR ligands and cytokines, CD141^hi^ DCs produce IFN-β, IFN-λ, and IL-12 (15, 24, 45) and can therefore participate in optimal Th induction. In addition, CD141^hi^ DCs are potent cross-presenting cells (11, 15) and they may play a role in T cell activation by presenting antigens from bystander-infected cells rather than by direct antigen presentation. Intriguingly, priming of CD4^+^ T cells in LNs of infected mice has been found to be mediated by non-infected CD11b^−^ DCs rather than Mtb-infected CD11b^+^ DCs (7). Whether this subset of CD11b^−^ DCs also comprise murine CD103^+^ DCs still needs to be addressed. In human TB a deeper analysis of CD141^hi^ DCs from LNs will be more informative in determining the relevance of CD141^hi^ and whether they cooperate with Mtb-infected myeloid DCs to activate a T cell response.

We show that contact between CD11c^+^ cells and pDCs occurs in LNs of TB patients. Furthermore, by using the specific pDC marker CLEC4C we identified the presence of GrB–pDCs, thus supporting previous data (46). GrB production in response to BCG was associated with enhanced IL-1β release by CD1c^+^ DCs and reduced bacterial growth (10). This phenotype was not observed in response to Mtb. Mtb activates type I IFN in macrophages and moDCs (47), and type I IFN has been shown to inhibit IL-1β production (48). Despite the low levels of IFN-α detected, it is possible that Mtb triggered the type I IFN pathway thereby counteracting the effect of pDCs on IL-1β release. Whereas GrB–pDCs alone acquire suppressive functions (49) we demonstrate here that they did not kill CD1c^+^ DCs; rather they strongly supported CD4^+^ T cell proliferation.

The presence of pDCs apparently did not affect activation of responding CD4^+^ T cells – at least at the gene transcript level analyzed here. Activated CD4^+^ T cells expressed transcripts of cytotoxic effector molecules such as perforin and GrB. Transcription factors for Th1, Th17, and Treg, but not Th2, cells were also upregulated. It has been shown that a large proportion of memory T cells in latent Mtb infection express a unique CXCR3^+^CCR6^+^ Th1 phenotype (50) but it remains unclear whether they are derived from Th1 or Th17 lineages. While we found that interplay of infected CD1c^+^ DCs and pDCs induced CD4^+^ T cell proliferation, further studies on activated CD4^+^ T cells and antigen specificity will determine their features and if they differentiate into memory T cells.

Taken together, these data suggest that communication between Mtb-susceptible and resistant DC subsets, plays an essential role in host defense to TB, thus calling for deeper investigations. Consequently, we propose that, while CD1c^+^ DCs are the more responsive DC subset to Mtb infection, pDCs help Mtb-infected CD1c^+^ DCs by intensifying stimulation during priming of naïve CD4^+^ T cells.

## Author Contributions

Laura Lozza designed and performed research, analyzed data, and wrote the paper. Maura Farinacci and Marina Bechtle contributed to experimental design, performed experiments and analysis, and revised the manuscript. Manuela Stäber and Ulrike Zedler performed experiments and analyzed data. Andrea Baiocchini and Franca del Nonno contribute to data acquisition, and revised the manuscript. Stefan H. E. Kaufmann supervised the study, contributed to experimental design, and writing of the paper.

## Conflict of Interest Statement

The authors declare that the research was conducted in the absence of any commercial or financial relationships that could be construed as a potential conflict of interest.
